# Serum Amyloid A: A Double-Edged Sword in Health and Disease

**DOI:** 10.3390/ijms26104528

**Published:** 2025-05-09

**Authors:** Ailing Ji, Luke W. Meredith, Preetha Shridas

**Affiliations:** 1 Saha Cardiovascular Research Center, University of Kentucky, 567 Charles T Wethington Building, 900 S. Limestone Street, Lexington, KY 40536-0200, USA; ailing.ji@uky.edu (A.J.); luke.meredith@uky.edu (L.W.M.); 2 Department of Internal Medicine, University of Kentucky, 567 Charles T Wethington Building, 900 S. Limestone Street, Lexington, KY 40536-0200, USA

**Keywords:** SAA, apolipoprotein, HDL, inflammation, lipid metabolism

## Abstract

Despite more than fifty years since its discovery in the 1970s, Serum Amyloid A (SAA)’s true biological functions remain enigmatic. The research so far has primarily associated SAA with chronic inflammatory conditions such as cardiovascular disease, obesity, and type 2 diabetes; its role in acute inflammation is less understood. Unlike the modest elevations observed in chronic conditions, SAA levels surge dramatically during acute inflammatory responses. Notably, approximately 2.5% of hepatic protein synthesis is devoted to SAA production during acute inflammation—despite the high energy demands required for synthesizing pro-inflammatory cytokines and immune cell activation—leaving its precise necessity unclear. Elucidating SAA’s physiological role in acute inflammation is crucial to determine the therapeutic potential of SAA inhibition for chronic inflammatory diseases, such as atherosclerosis and abdominal aortic aneurysms. The evidence suggests that SAA may play a protective role in acute inflammation, positioning it as a “double-edged sword”: detrimental in chronic inflammation, yet potentially beneficial in acute settings. This review explores the divergent roles of SAA in chronic versus acute inflammation, proposing that while SAA inhibition could offer therapeutic benefits for chronic conditions, it might pose risks during acute inflammation. As the primary transporter of SAA in circulation, high-density lipoprotein (HDL) has been shown to become dysfunctional in chronic inflammation, at least partly due to SAA’s effects. However, we propose that SAA may confer functional properties to HDL during acute inflammatory states, such as sepsis, thereby highlighting the context-dependent nature of its impact.

## 1. Introduction

Serum Amyloid A (SAA) consists of a family of small proteins (104–112 amino acids) that share high levels of sequence homology [1]. First isolated in the 1960s from the spleens and livers of individuals with secondary amyloidosis [2], SAA proteins are encoded by separate but closely related genes that have been remarkably conserved throughout vertebrate evolution [3]. In humans, the SAA gene cluster is located on the short arm of chromosome 11, within a 150 Kb area, in the p15.1 region [4,5]. There are four SAA genes (*SAA1*, *SAA2*, *SAA3*, and *SAA4*) in humans. *SAA1* and *SAA2* encode acute-phase SAA proteins highly inducible during the acute-phase response. SAA4 is a constitutively expressed SAA protein, and *SAA3* is a pseudogene [3]. Mice also have four SAA genes, similar to humans. *Saa1.1* and *Saa2.1* encode acute-phase proteins, and *Saa4* codes for a constitutively expressed SAA. Unlike human *SAA3*, *Saa3* in mice encodes a functional product [6,7,8]. SAA4 also has N-glycosylation sites towards its C-terminal end and circulates in glycosylated and non-glycosylated forms [2]. SAA4 is likely the major isoform in the circulation of healthy individuals; however, very little is known about its biological properties. All genes share the same organization of four exons and three introns. An 18 aa signal sequence is present in the initial transcript but is removed in the serum proteins [9]. SAA subtypes have been highly conserved through evolution [10]. Murine SAA1 and SAA2 share 96% sequence homology, while SAA3 is 63% and 65% homologous to SAA1 and SAA2, respectively [11].

SAA structure is characterized by four alpha-helices that form a cone-shaped bundle, with the C-terminal residues wrapping around the bundle [12,13]. Due to the hydrophobic nature of helices 1 and 3, the SAA monomer exhibits poor solubility. Multiple oligomeric forms of SAA have been reported, including hexameric SAA1 [13], and a soluble SAA octamer that transitions to a hexameric species over time [14]. Nady et al. (2024) reported the presence of soluble SAA octamers with structural similarity to the ring-like formations observed in lipid-free ApoA-I [15]. These SAA octamers can scaffold lipid nanodiscs with a morphology similar to those formed by ApoA-I. The SAA–lipid nanodiscs, containing four SAA molecules, suggest a novel model for SAA–lipid interactions [15]. Murine SAA3 is reported to exist as a tetramer, with its core predicted to serve as the binding site for retinal [16].

## 2. Expression Profile

SAA1/2 is highly expressed in the liver in response to inflammatory stimuli, such as tissue injury, infection, and trauma, and once secreted into the circulation, it becomes a major apolipoprotein of high-density lipoprotein (HDL) [17]. SAA1/2 is also synthesized in various other tissues, including the breast, stomach, small and large intestine, prostate, lung, pancreas, kidney, tonsil, thyroid, pituitary, placenta, skin epidermis, and brain neurons [18]. SAA3 is expressed in the liver and extrahepatic tissues, including adipose tissue, lung, kidney, adrenal gland, small intestine, ileum, heart, and macrophages [6,19,20]. Like SAA1/2, SAA3 is present in the circulation bound to HDL, although to a lesser level than SAA1/2. However, unlike SAA1.1/2.1, a portion of SAA3 in plasma is present in a lipid-free form [20]. Most cell types constitutively express SAA4 and exhibit a minimal response to inflammatory stimuli [1,21].

## 3. Regulation of SAA Gene Expression

Acute-phase *SAA* promoters contain NF-κB and C/EBP transcription factor recognition sequences, which play critical roles in cytokine responsiveness and cell-specific gene expression [1,22,23]. The principal cytokines involved in the induction of acute-phase SAA are IL-1, TNF-α, and IL-6 [1]. Additional cytokines that may directly or indirectly contribute to SAA induction include IL-2, interferon-γ (IFN-γ), and ciliary neurotrophic factor [24,25,26]. Glucocorticoids, released during inflammation, have also been shown to enhance cytokine-induced SAA expression [1]. A study in mice demonstrated that vitamin A regulates Saa gene expression [7]. Further, Derebe et al. showed reduced *Saa1* and *Saa2* transcription in the intestines of mice fed a vitamin A-deficient diet. Similarly, in HepG2 cells treated with both IL-1β and IL-6, the addition of retinol or retinoic acid further enhanced Saa1 expression [16].

## 4. SAA Receptors and Functions

SAA is known to interact with a variety of receptors, primarily based on in vitro studies. Using primary mouse macrophages and macrophage-like cell lines, Lee et al. showed that SAA contributes to macrophage foam cell formation via formyl peptide receptor 2 (FPR2) signaling [27]. SAA has been shown to induce IL-1β and IL-6 production in macrophages, partially dependent on ALX/FPR2 signaling [28]. SAA has also been reported to promote proliferative and pro-inflammatory responses in keratinocytes through FPR2-dependent mechanism [29]. SAA stimulated CCL2 expression in human umbilical vein endothelial cells (HUVECs) via a pertussis toxin (PTX)-sensitive pathway and induced the activation of three MAP kinases (ERK, p38 MAPK, and JNK), which was inhibited by FPR2 silencing [30]. SAA inhibited human neutrophil apoptosis through a P2X7-sensitive pathway [31]. SAA has been shown to act as a ligand for receptor for advanced glycation end products (RAGE). SAA induced tissue factor (TF) expression in human monocytes; approximately 50% of TF expression was inhibited when treated with a RAGE competitor, soluble RAGE, and by about 85% following treatment with anti-RAGE IgG, indicating the role of RAGE [32]. RAGE, a receptor for the amyloidogenic form of SAA, is upregulated in systemic amyloidosis, particularly in amyloid-laden spleens. In these spleens, RAGE is predominantly expressed by mononuclear phagocytes. Additionally, monocytes/macrophages in affected spleens strongly express the M-CSF antigen, and splenic tissue with deposited amyloid A exhibits increased IL-6 expression [33]. Rheumatoid arthritis (RA) is a chronic, symmetric, polyarticular joint disease characterized by inflammation. SAA is significantly upregulated during RA progression and binds to the receptor for RAGE, activating the NF-κB signaling pathway in rheumatoid synovial fibroblasts. This SAA-RAGE-induced NF-κB activation plays a pivotal role in RA pathogenesis [34]. SAA is a ligand for scavenger receptor class B type I (SR-BI) and inhibits HDL binding and selective lipid uptake in Chinese hamster ovary (CHO) cells expressing SR-BI [35]. SAA binding to SR-BI mediates the activation of ERK1/2 and p38 MAPK in both SR-BI-overexpressing HeLa cells and the THP-1 monocyte cell line [36]. The SAA-SR-BI-mediated p38 MAPK signaling pathway has been implicated in RA-associated angiogenesis in HUVECs [37]. CD36, a class B scavenger receptor expressed on phagocytes, serves as a receptor for SAA, mediating its pro-inflammatory activity via JNK- and ERK1/2-dependent signaling pathways [38].

The functions of SAA have been associated with its interactions with Toll-like receptors (TLRs), specifically TLR2 [39] and TLR4 [40]. SAA stimulation of these receptors triggers MyD88-dependent signaling, leading to the phosphorylation of ERK and p38 MAPK, as well as transcriptional activation that enhances the expression of TNF-α, IL-12p40, IL-1 receptor antagonist (IL-1rn), IL-10 and IL-23p19 [39], and elevated production of NO [40]. However, some of its interactions, particularly with TLRs 2 and 4, have been contested as true SAA receptors due to potential bacterial contaminants in recombinant SAA preparations that could activate these receptors [41,42]. Thus, several in vitro studies have demonstrated that SAA induces pro-inflammatory responses through multiple cell surface receptors; however, these findings have yet to be validated in vivo.

## 5. Serum Amyloid A-Mediated Regulation of Inflammatory Cell Migration and Recruitment

**Figure 1:**
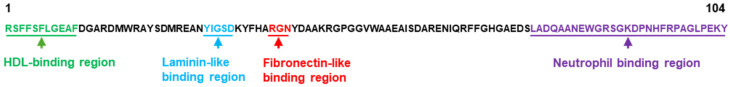
HDL- and leukocyte-binding motifs within the primary structure of human SAA1 (mature protein is 104 amino acids long [41].

Badolato et al. reported that recombinant human SAA (rhSAA) induces directional migration of monocytes and polymorphonuclear leukocytes (PMNs), modulates the expression of adhesion proteins CD11b and leukocyte cell adhesion molecule I, and enhances the adhesion of PMNs and monocytes to umbilical cord vein endothelial cell monolayers. Subcutaneous injection of rhSAA in mice resulted in the recruitment of PMNs and monocytes to the injection site [43]. rhSAA also induced the directional migration of T cells in vitro [44]. The T cell chemotaxis triggered by rhSAA was inhibited by pretreatment with pertussis toxin, indicating its interaction with a G-protein-coupled receptor. Additionally, subcutaneous administration of rhSAA into huPBL-SCID mice led to the infiltration of human T lymphocytes at the injection sites [44]. SAA selectively induced Ca^2+^ mobilization and migration in human embryonic kidney (HEK) cells expressing the G protein-coupled receptor formyl peptide receptor-like 1 (FPRL1), a human seven-transmembrane domain phagocyte receptor with low affinity for fMLP and high affinity for lipoxin A4. Thus, SAA was identified as the first chemotactic ligand for FPRL1 [45]. SAA and Cxcl1/KC were reported to promote the mobilization of myeloid-derived suppressor cells cooperatively by a pathway dependent on hepatic IL-6-gp130-STAT3 signaling in the liver during sepsis [46]. Recombinant human SAA has exhibited chemoattractant properties for several immune cells, including human monocytes, neutrophils, and T lymphocytes in vitro and in vivo in mice through FPRL1 [43,44,45]. Consistent with these findings, Anthony et al. [47] demonstrated that SAA induces neutrophil recruitment in chronic obstructive pulmonary disease lungs in an IL-17A-dependent manner. Although several reports, primarily using recombinant SAA preparations, have shown that SAA promotes immune cell chemotaxis and tissue recruitment, studies utilizing SAA-deficient or overexpressing mouse models suggest that SAA may instead suppress neutrophil recruitment to injured organs during acute inflammation. Ji et al. [48] reported that deficiency of SAA significantly increased myeloperoxidase activity and neutrophil counts in the injured lung 24 h after the induction of sepsis in mice; consistent observations were reported by Cheng et al. [49] and Fan et al. [50]. Interestingly, SAA, whether free or lipid-bound, can specifically bind to neutrophils and is thought to modulate their migration across endothelial barriers from the circulation to sites of inflammation [51]. Synthetic peptides related to amino acid residues 29–42 of human SAA were found to inhibit the adhesion of human T-lymphocytes and mouse M4 melanoma cells to the glycoproteins of the extracellular matrix, laminin, or fibronectin [52]. The HDL- and leukocyte-binding motifs in the primary structure of human SAA1 are depicted in Figure 1.

## 6. Pro-Inflammatory Properties of SAA

As discussed earlier, a substantial body of in vitro studies suggests that SAA functions as a pro-inflammatory molecule. Consequently, it has long been widely believed that SAA plays a central role in orchestrating inflammatory responses during acute infections or tissue injuries [53]. Recombinant human SAA has been shown to exhibit cytokine-like properties, inducing the synthesis of IL-1β, IL-1 receptor antagonist (IL-1ra), and sTNFR-II at both the protein and mRNA levels in THP-1 cells [54]. Additionally, it enhanced the release of TNF-α, IL-1β, and IL-8 by human blood neutrophils [55]. Human neutrophils secreted the pro-inflammatory cytokine interleukin-8 (IL-8) in response to SAA through the activation of the FPRL1 receptor [56]. Local expression of Serum Amyloid A and FPRL1 genes in synovial tissue is associated with the production of matrix metalloproteinases (MMPs) in patients with inflammatory arthritis [57]. SAA potently induced TF expression and IL-12p40 secretion in monocytes [32,58]. SAA is shown to be a potent endogenous stimulator of granulocyte colony-stimulating factor (G-CSF), a key cytokine regulating granulocytosis [59]. SAA proteins have been shown to cross the blood–brain barrier and impair its function [2].

In addition to its pro-inflammatory effects predominantly observed in vitro, several reports have paradoxically suggested that SAA may exhibit anti-inflammatory properties, either directly or indirectly. Ex vivo and in vitro stimulation of human blood monocytes and macrophages with recombinant human SAA induced the release of M2 macrophage markers, including IL-10, arginase 1, and Ym1, through a MyD88-dependent mechanism [60]. Using a transgenic mouse model that overexpresses human SAA1 in macrophages, Cheng et al. reported that SAA suppresses lipopolysaccharide-induced pro-inflammatory responses by promoting its clearance from the circulation through direct binding [49]. Nguyen et al. reported that SAA induced the proliferation of the tolerogenic subset of regulatory T cells, leading to their expansion in the micro-environment at sites of infection or tissue injury, thereby suppressing inflammation [61]. Sander et al. demonstrated SAA’s role in suppressing the pro-inflammatory response by mobilizing myeloid-derived suppressor cells [46].

## 7. The Controversy: Is Serum Amyloid Truly Pro-Inflammatory?

Many in vitro functional studies of SAA have utilized recombinant preparations; however, comparisons between recombinant and endogenous SAA have demonstrated distinct differences in their properties; added to this problem, some of the properties are attributed to endotoxin contamination in bacterial-derived recombinant SAA [62]. Transgenic overexpression of SAA in the liver did not trigger a systemic inflammatory response, even though it resulted in a significant increase in SAA in plasma to levels typically associated with a robust acute phase response (i.e., >1 mg/mL) [63]. Additionally, studies by Ji et al. [48] reported no differences in systemic inflammatory markers with SAA deficiency in a mouse model of sepsis. The above observations raise an important question regarding whether SAA is truly a pro-inflammatory molecule.

Many of the pro-inflammatory activities of SAA observed in vitro are lost when SAA is bound to HDL [64,65]. For instance, stimulation of mouse monocyte J774 cells with lipid-poor rhSAA and purified endogenous SAA from cardiac surgery patients elicited the production of pro-inflammatory cytokines, such as G-CSF. In contrast, HDL-associated SAA did not stimulate the production of these cytokines [64]. Furthermore, SAA-induced TNF-α production was higher in medium containing lipoprotein-deficient serum compared to medium containing normal human serum. The addition of HDL inhibited SAA-induced TNF-α release in a dose-dependent manner. This inhibitory effect was specific to HDL and was not influenced by low-density lipoprotein (LDL) or very-low-density lipoprotein (VLDL). A similar inhibitory effect was also observed for IL-1β release [66]. SAA purified from acute-phase mouse plasma, stimulated IL-1β secretion in murine J774 cells and bone marrow-derived macrophages through a mechanism dependent on NLRP3 expression and caspase-1 activity [65]. Notably, incorporating SAA into HDL prior to cell treatments completely abolished its ability to stimulate ROS generation and inflammasome activation. These findings underscore HDL’s crucial role in modulating SAA’s pro-inflammatory effects [65]. Virtually all SAA in plasma is associated with HDL [20]; 95% of the liver-derived SAA exists bound to HDL in circulation [17], which raises an interesting question about the activities of circulating SAA in vivo.

## 8. Results from Animal Models of SAA

With the development of knockout and transgenic mouse models of SAA, several in vivo functions of SAA have come to light. SAA is chronically elevated, although at more modest levels, in individuals with chronic inflammatory conditions such as rheumatoid arthritis [57,67], atherosclerosis [68,69,70], abdominal aortic aneurysms [71,72], cancer metastasis [73,74], Crohn’s disease [75] and type 2 diabetes [76]. Chronic elevation of SAA can lead to the formation of amyloid fibrils, resulting in amyloid A (AA) amyloidosis. This occurs through a process of cleavage, misfolding, and aggregation into a highly ordered abnormal β-sheet conformation, with the resulting fibrils depositing in tissues and organs, ultimately causing dysfunction [77,78,79,80]. Antisense oligonucleotide-mediated suppression of SAA reduced amyloid deposition in mice with AA amyloidosis [81]. Using dose-dependent, doxycycline-inducible transgenic expression of SAA in mice, it was demonstrated that AA amyloid deposition can occur independently of inflammation and that it is dependent on the circulating SAA concentration [63].

SAA levels are chronically elevated in cancer. A recent study by Lee et al. [73] showed an important role played by SAA in developing a pro-metastatic niche leading to liver metastasis. Mice deficient in SAA failed to show features of pro-metastatic niche in the liver and for fibrosis and myeloid cell recruitment, indicating the importance of SAA in this process. Production of SAA through the activation of IL-6-STAT3 signaling was shown to alter the immune and fibrotic microenvironment of the liver to establish a pro-metastatic niche.

Chronic elevation of SAA is found in humans with cardiovascular disease (CVD), including abdominal aortic aneurysms (AAA) and atherosclerosis [82,83] and increased circulating SAA is associated with CVD mortality [84]. The pro-inflammatory and pro-thrombotic activities of SAA are thought to contribute to the development and progression of atherogenesis and atherothrombosis [85]. SAA is deposited in atherosclerotic lesions of low-density lipoprotein receptor-deficient (LDLR-/-) and apolipoprotein E-deficient (apoE-/-) mice at all stages of lesion development, with SAA immunoreactive areas strongly correlating with lesion size [86]. Lentivirus-mediated overexpression of SAA1 in male apoE-/- mice resulted in increased inflammatory cell infiltration and atherosclerotic lesion development [70]. SAA administration in apoE-/- mice led to vascular inflammation, enhanced formation of atherosclerotic lesion, and renal dysfunction. Even a single injection of the adenoviral vector encoding SAA1, resulting in only a brief elevation of circulating SAA, was sufficient to increase atherosclerosis in apoE-/- mice in the immune-tolerant recombination activating gene-1-deficient background [87]. However, no reduction in atherosclerosis was observed in the absence of endogenous SAA1.1 and SAA2.1 in apoE-/- (DKO) mice [88], while suppression of SAA3 in DKO mice significantly reduced atherosclerosis [89]. These results suggest that all acute-phase SAA isoforms possess pro-atherogenic properties, indicating that suppressing all three SAA isoforms may be necessary for effective atheroprotection. Cai et al. [90] reported that i.p. injection of recombinant human SAA in apoE-/- promoted endothelial inflammation and dysfunction, which was associated with cardiovascular disease and renal pathologies; however, these effects were not observed when SAA was injected with HDL [90].

ApoE-/- mice deficient in SAA are protected from angiotensin II (AngII)-induced AAA development. SAA colocalized with breaks in the elastin lamina, prominent MMP activity, and macrophages in the aneurysmal tissue of apoE-/- mice chronically infused with AngII [72]. Using mice with highly inducible SAA expression in adipose tissue, it was demonstrated that adipocyte-specific SAA expression alone can induce AAA in obese mice infused with AngII, which was associated with increased MMP activity and macrophage infiltration in the abdominal aortas of these mice [91]. Furthermore, suppression of SAA with antisense oligonucleotide (SAA-ASO) limited AngII-induced AAA progression in obese C57BL/6 mice, accompanied by significant reductions in MMP activities, decreased macrophage infiltration, and fewer elastin breaks in the abdominal aortas [71].

**Figure 2:**
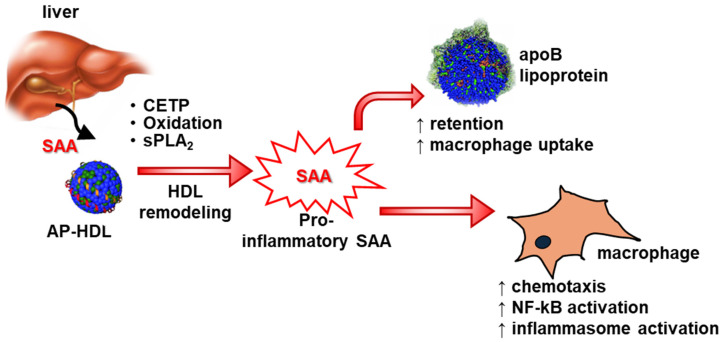
Proposed scheme for SAA’s role in atherosclerosis. During chronic inflammation, HDL remodeling is facilitated by proteins such as CETP, which release lipid-free or lipid-poor SAA, which then exerts its pro-atherogenic effects [27,39,43,44,45,65,92,93,94,95]. AP-HDL, acute-phase HDL; CETP, cholesteryl ester transfer protein; sPLA_2_, secretory phospholipase A_2_. ↑ Indicates enhancement of the process.

As described earlier, HDL masks the pro-inflammatory effects of SAA in vitro, and more than 98% of SAA remains HDL-bound in circulation [17]. Transgenic overexpression of SAA in the liver, which increased plasma SAA levels more than 1 mg/mL, did not alter pro-inflammatory responses during acute inflammation in vivo [63]. However, as mentioned in the previous paragraph, SAA has been shown to stimulate pro-inflammatory responses in vivo under certain pathological conditions. For example, mice deficient in SAA demonstrated significantly lower expression of TGF-β and matrix metalloproteinase-2 expression in the aorta following Ang-II infusion [72]. Consistently, AngII infusion resulted in a significant reduction in plasma IL-1β levels in apoE-/- mice deficient in SAA compared to wild type mice [65]. These reports may indicate that under certain pathological conditions, SAA may become HDL-free and possibly trigger pro-inflammatory responses. Cholesteryl ester transfer protein (CETP)-mediated remodeling of SAA-enriched HDL led to the release of lipid-poor SAA [92]. While HDL-bound SAA (SAA-HDL) did not stimulate IL-1β release from J774 cells [65], CETP remodeling of SAA-HDL liberated free SAA from HDL and, interestingly, triggered IL-1β release by these cells [93]. Consistently, CETP expression increased atherosclerosis in apoE-/- mice and enhanced SAA deposition in the aortic atherosclerotic lesions; however, there was no change in lesion development in apoE-/- mice deficient in SAA regardless of CETP expression [93]. Thus, these results may indicate that lipid-free SAA is pro-inflammatory, and any factor such as CETP that liberates SAA from HDL may promote pro-inflammatory responses (Figure 2).

While the modest elevation of circulating SAA during chronic inflammatory diseases such as atherosclerosis and abdominal aortic aneurysm promotes inflammation, leading to the development of the diseases, most reports indicate that SAA is protective during acute inflammatory diseases such as sepsis [48,49,96]. Sander et al. [46] reported that SAA produced in the liver by hepatic IL6-gp130-STAT3 activation promoted anti-inflammatory properties by mobilizing myeloid-derived suppressor cells into the injured liver during sepsis, thus promoting survival. In a dextran sulfate sodium (DSS)-induced colitis mouse model, SAA3 deficiency exacerbated the disease condition by increasing the expression of pro-inflammatory cytokines and decreasing the expression of IL-22-producing neutrophils [97]. Using mice with inducible transgenic expression of human SAA1, Cheng et al. demonstrated that SAA1 acts as an LPS-binding protein, assisting in the clearance of endotoxins from circulation and thereby reducing systemic inflammatory responses [49]. The same group showed that the genetic deletion of Saa3 increased susceptibility to Pseudomonas aeruginosa infection in mice, leading to exacerbated inflammatory responses and more pronounced neutrophil infiltration in the lungs [50]. Mice deficient in all three acute-phase isoforms of SAA showed that the deficiency of endogenous SAA worsens mortality across three sepsis models: cecal ligation and puncture (CLP), cecal slurry treatment, and endotoxemia [48]. Endogenous SAA is thus essential for sepsis survival. Several reports indicate that SAA transports retinol during acute inflammation [16,98]. In a recent report [99], Bang et al. showed that SAA transports retinol to myeloid cells during acute inflammation promoted by Salmonella typhimurium infection. They further showed that SAA-retinol complex is endocytosed and metabolized by myeloid cells in an LDL receptor-related protein 1 (LRP1)-dependent manner; this process is critical for B and T cell development and homing to the gut and immunoglobulin A production by B cells. Thus, the report indicated a new role for SAA in conferring vitamin A-mediated immunity.

While most animal studies have been conducted in mice, relatively few have utilized other species; such studies were mainly based on SAA as a biomarker in disease conditions. SAA is recognized as a major acute-phase protein in swine [100]. In experimental models, lung infection induced by the pig-specific respiratory pathogen *Actinobacillus pleuropneumoniae* resulted in a pronounced increase in SAA levels within 14–18 h post-infection. This response involved both hepatic and extrahepatic production of SAA, indicating a systemic acute-phase reaction [101]. Notably, the duration of this response was significantly reduced by antimicrobial treatment, underscoring the potential of SAA as a valuable biomarker for monitoring therapeutic outcomes [102]. Beyond infectious disease, SAA has also been investigated as a biomarker of stress in swine. Salivary SAA concentrations rose markedly following road transport, with peak levels observed upon arrival at the slaughterhouse and during lairage at 30 and 60 min. These findings support the use of salivary SAA as a non-invasive marker of complex stress in pigs [103]. In rabbits fed a high-cholesterol diet and treated with apolipoprotein A-I mimetic peptides, plasma SAA levels—but not total cholesterol levels—were positively associated with the extent of the atherosclerotic lesion area [104]. In a rabbit model of atherosclerosis, stent implantation triggered an acute phase response including elevated SAA levels, which were associated with increased plaque burden and features of plaque instability in the progression of nontarget lesions [105].

## 9. SAA’s Impact on HDL’s Properties

The presence of HDL is essential for the existence of SAA in circulation, as evidenced by the dynamic and sharp increase in SAA levels during the acute-phase response, which would not occur without HDL [94]. The ability of HDL to mitigate the pro-inflammatory effects of SAA was previously discussed. Here, we explore how SAA influences HDL’s functional properties. In vitro studies using adipocytes and macrophages demonstrated that HDL exhibits anti-inflammatory effects, which are lost when enriched with SAA [106]. HDL from SAA-deficient mice showed enhanced anti-inflammatory properties in vitro compared to HDL from wild-type mice with circulating SAA [107]. Additionally, HDL’s anti-inflammatory properties were inversely correlated with SAA levels in patients with end-stage renal disease. In a large cohort of high cardiovascular risk patients, in vivo and in vitro evidence revealed that SAA transforms HDL from an atheroprotective to a pro-inflammatory lipoprotein [108]. Among these patients, elevated SAA levels were associated with increased mortality despite higher HDL-C, while lower SAA concentrations linked HDL-C to reduced all-cause and cardiovascular mortality. Experimental studies further showed that SAA-enriched HDL reduces endothelial nitric oxide production while increasing reactive oxygen species [108]. These findings suggest that SAA modifies HDL’s vascular properties, rendering it dysfunctional. Thus, in conditions such as coronary artery disease, diabetes, smoking, aging, and renal impairment—where SAA levels are modestly elevated—HDL is considered to be dysfunctional. This transformation from “functional to dysfunctional” HDL with SAA association underscores the potential detrimental effects of raising HDL concentrations in patients with high SAA levels in patients with chronic inflammatory diseases such as coronary artery diseases.

However, contrasting findings exist, with reports indicating no significant effects of SAA on HDL’s anti-inflammatory properties [109]. Interestingly, a study comparing SAA-free HDL from patients before cardiac surgery and SAA-enriched HDL from the same patients three days post-surgery found no significant differences in suppressing TNF-α-induced vascular cell adhesion molecule-1 expression in endothelial cells [110]. In another study, SAA was reported to promote the antioxidant ability of HDL [111]; other studies have also supported this. Jayaraman et al. [112] reported that adding SAA reduces the oxidation of HDL and LDL lipids. These findings highlight the complexity of SAA’s effects on HDL functionality and underscore the need for further research.

HDL levels decrease significantly during acute inflammation, with clinical data showing a reduction of 40–70% in septic patients, which is associated with poor prognosis. Several studies report that survivors of acute inflammation tend to have higher HDL cholesterol levels compared to nonsurvivors [95,113,114,115,116,117]. Experimental therapies aimed at elevating HDL levels have demonstrated improved sepsis survival in animal models [94,118].

SAA is a key apolipoprotein associated with HDL during acute inflammation, including sepsis [17,119]. During the inflammatory response, SAA replaces traditional apolipoproteins on HDL, with nearly every HDL particle incorporating at least one SAA molecule [17,120]. Interestingly, the dramatic rise in SAA levels observed during acute inflammation does not occur in mice lacking HDL [94]. This suggests that HDL is essential for the presence of SAA in circulation and that the marked increase in SAA during the acute-phase response depends on HDL availability. Given that proteins constitute the primary structural and functional components of HDL, and SAA, a major protein on HDL during acute inflammation, the protective role of SAA raises a critical question: does SAA contribute to the protective properties of HDL during acute inflammation?

## 10. Conclusions

SAA remains an enigmatic protein. Despite being discovered over 50 years ago, its biological functions are not yet fully understood. Several factors contribute to this knowledge gap, including the inability to replicate findings from studies using recombinant SAA with properties of endogenous protein, discrepancies in findings between in vitro and in vivo experiments, and variations in its functionality when bound to HDL versus in its free form. Thus, findings from in vitro studies must be critically evaluated and supported by in vivo evidence to ensure their biological relevance.

**Figure 3:**
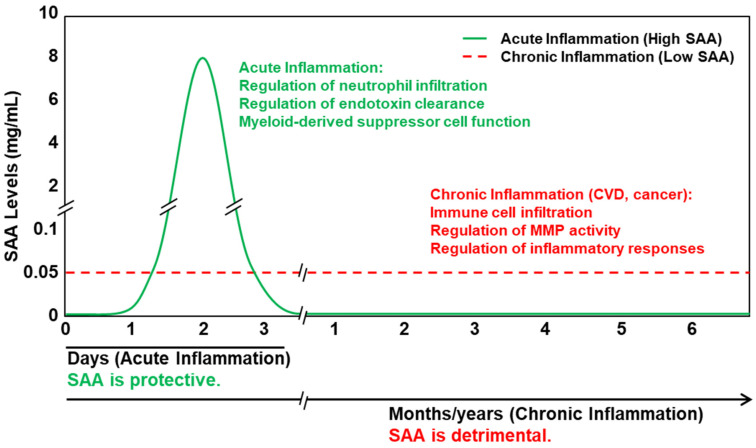
Dynamics of SAA in plasma with approximate levels and known roles during acute and chronic inflammation [46,48,49,50,70,71,72,74,91,97].

Based on available data from validated in vitro properties of SAA and animal studies in vivo, we hypothesize that Serum Amyloid A (SAA) functions as a double-edged sword. During acute inflammation, transient and dramatic elevations in SAA are protective. In contrast, under chronic inflammatory conditions, dysregulated and sustained—albeit modest—increases in SAA contribute to disease progression, as observed in disorders such as cardiovascular disease and cancer (Figure 3). We propose that HDL plays a critical role in modulating the functional properties of SAA; when SAA is bound to HDL, its pro-inflammatory properties are at least partially masked.

HDL plays a critical role in SAA’s functionality. While several publications document that HDL masks several pro-inflammatory properties of SAA and that SAA contributes to HDL dysfunction in CVD, the potential role of SAA in imparting beneficial, protective properties remains unexplored.

## Abbreviations

AA, amyloid A; AAA, abdominal aortic aneurysms; AngII, angiotensin II; apoE-/-, apolipoprotein E-deficient mice; CETP, cholesteryl ester transfer protein; CHO, Chinese hamster ovary; CLP, cecal ligation and puncture; CVD, cardiovascular disease; FPR2, formyl peptide receptor 2; FPRL1, formyl peptide receptor-like 1; G-CSF, granulocyte colony-stimulating factor; HDL, high-density lipoprotein; HEK, human embryonic kidney; HUVECs, human umbilical vein endothelial cells; IFN-γ, interferon-γ; LDL, low-density lipoprotein; LDLR-/-, low-density lipoprotein receptor-deficient mice; IL-1rn, IL-1 receptor antagonist; LRP1, LDL receptor-related protein 1; MMPs, matrix metalloproteinases; PMNs, polymorphonuclear leukocytes; PTX, pertussis toxin; RA, Rheumatoid arthritis; RAGE, receptor for advanced glycation end products; SAA, Serum Amyloid A; rhSAA, recombinant human SAA; SR-BI, scavenger receptor class B type I; TF, tissue factor; TLRs, Toll-like receptors; VLDL, very-low-density lipoprotein.
